# PROTEIN BIOMARKERS, CELLULAR SENESCENCE AND SECRETOMES, EXOSOMES AND BIOFLUIDS

**DOI:** 10.1093/geroni/igae098.0983

**Published:** 2024-12-31

**Authors:** Birgit Schilling, Melanie Koenigshoff

**Affiliations:** Buck Institute for Research on Aging, Novato, California, United States; University of Pittsburgh, Pittsburgh, Pennsylvania, United States

## Abstract

In this component of the session, the opportunities and challenges of biomarker discovery and validation will be discussed and involve the audience. Cellular senescence-related biomarkers have been shown to be good biomarker candidates for aging. Key questions that will be raised will include organ-specific biomarkers, and the use and culturing of explant human tissues to generate specific biomarkers of senescence and aging. We will discuss the senescence-associated secretory phenotype (SASP), the role of the extracellular matrix during aging and their use as aging biomarkers. We will discuss challenges of biofluid complexities and methods to address these challenges. We will also discuss the value of multi-omics biomarkers, such as proteins, transcripts and lipids.

